# FOXP1-induced lncRNA CLRN1-AS1 acts as a tumor suppressor in pituitary prolactinoma by repressing the autophagy via inactivating Wnt/β-catenin signaling pathway

**DOI:** 10.1038/s41419-019-1694-y

**Published:** 2019-06-24

**Authors:** Chao Wang, Chunlei Tan, Yuan Wen, Dongzhi Zhang, Guofu Li, Liang Chang, Jun Su, Xin Wang

**Affiliations:** Department of Neurosurgery, The Affiliated Cancer Hospital of Harbin Medical University, No.150, Haping Road, Nangang, Harbin, 150001 Heilongjiang China

**Keywords:** Cancer, Molecular biology

## Abstract

As the commonest type of functional pituitary tumor, prolactinoma takes up around 40–60% of functional pituitary tumors. Despite dedications attributed to the treatment of prolactinoma, complete cure remains difficult. Hence, it is of significance to bring to light the underlying mechanism of prolactinoma. Long noncoding RNAs (lncRNAs) are a group of transcripts which can regulate various biological processes. In the present study, we explored an lncRNA that was differentially downregulated in prolactinoma samples. LncRNA clarin 1 antisense RNA 1 (CLRN1-AS1) was downregulated in 42 patient samples and inactivated the Wnt/β-catenin signaling pathway. Functionally, CLRN1-AS1 suppressed cell proliferation, promoted apoptosis, and inhibited autophagy. Subcellular fractionation assay revealed that CLRN1-AS1 was located in the cytoplasm of prolactinoma cells. Based on bioinformatics analysis and mechanism experiments, we determined that CLRN1-AS1 acted as a competing endogenous RNA (ceRNA) by sponging miR-217 to upregulate the dickkopf WNT signaling pathway inhibitor 1 (DKK1). Furthermore, Forkhead box P1 (FOXP1) was verified to be a transcription suppressor of CLRN1-AS1. In summary, this study revealed that FOXP1-induced CLRN1-AS1 regulated cellular functions in pituitary prolactinoma by sponging miR-217 to release the DKK1/Wnt/β-catenin signaling pathway.

## Introduction

Pituitary adenoma, the second commonest cancer in the central nervous system which takes up 14% in intracranial neoplasm, has an overall prevalence around 1/1500 persons^1,2^. Among pituitary adenomas, pituitary prolactinoma is the most prevalent type, occupying about 40–60% of all functional pituitary tumors^3^. Therefore, exploring the molecular mechanism involved in the initiation or progression of pituitary prolactinoma is of great importance to new direction for the treatment of pituitary prolactinoma.

Long noncoding RNAs (lncRNAs) are a group of long transcripts (>200 nucleotides) without protein-coding ability^4^. LncRNAs have been identified as participants in various cellular, physiological, and pathological processes through a number of regulatory mechanisms^5–7^. Dysregulation of lncRNAs in malignancies can promote or suppress tumorigenesis^8–11^. Recent years, some researchers reported that lncRNAs can exert tumor suppressive or oncogenic roles in pituitary prolactinoma^12–14^. Nevertheless, the mechanism and function of lncRNAs in pituitary prolactinoma remain largely unknown. The aim of this study is to explore the function and molecular mechanism of a novel lncRNA in pituitary prolactinoma. Through microarray analysis, we determined that lncRNA clarin 1 antisense RNA 1 (CLRN1-AS1) was significantly downregulated in pituitary prolactinoma samples.

The Wnt/β-catenin signaling pathway is a crucial participant in tumor progression^15–17^. In our present study, we explored the potential regulatory mechanism between the CLRN1-AS1 and Wnt/β-catenin signaling pathway. LncRNAs are acknowledged as a group of transcripts that can posttranscriptionally regulate miRNAs to release the downstream mRNAs^18–20^. In our present study, we identified the cytoplasmic localization of CLRN1-AS1 in pituitary prolactinoma cell lines, which prompted us to detect its downstream miRNA and mRNA. Bioinformatics analysis and mechanism experiments were used to demonstrate that CLRN1-AS1 can act as a competing endogenous RNA (ceRNA) to upregulate the dickkopf WNT signaling pathway inhibitor 1 (DKK1) by sponging miR-217. Moreover, the upstream molecular mechanism of CLRN1-AS1 was analyzed. Forkhead box P1 (FOXP1) was found to be a transcription suppressor of CLRN1-AS1. Taken together, this study focused on the regulatory mechanism between the CLRN1-AS1 and DKK1/Wnt/β-catenin signaling pathway, thereby elucidating a novel molecular pathway in pituitary prolactinoma.

## Materials and methods

### Tissue specimen

Tumor tissues and adjacent normal tissues were obtained from The Affiliated Cancer Hospital of Harbin Medical University. All patients had been definitely diagnosed with pituitary prolactinoma by imaging, surgery, and pathological examination. None of the patients were treated with any therapy before surgery. Every patient enrolled in this study has provided written informed consent. This study has been ethically permitted by the ethics committee of The Affiliated Cancer Hospital of Harbin Medical University. Tissue samples were immediately frozen in liquid nitrogen as soon as it was extracted from patients and stored at −80 °C until extraction of RNAs.

### Primary pituitary prolactinoma cell (PPA) culture

PPA culture was conducted in a human solid biopsy of a patient with pituitary prolactinoma who was diagnosed at The Affiliated Cancer Hospital of Harbin Medical University. To remove the adhering blood and visible necrotic portions, the fresh tumor biopsies were carefully washed. Then, the samples were sliced into small pieces (1 mm^3^) and washed twice with DMEM serum-free solution. Next, the tissue specimens were incubated with 0.125% trypsin and 0.125% EDTA (pH 7.4). The ratio between the weight of pituitary prolactinoma tissue and trypsin was 1 g/10 ml. Digestion was conducted at 37 °C for about 20 min in a water bath via gentle stirring. PPA cell was obtained by centrifugation and grown in adherent and neurosphere conditions. For adherence, cells were plated in a tissue culture flasks (75 cm^2^), suspended in DMEM with 10% FBS. Cells were incubated at 37 °C with 5% CO_2_.

### Cell lines and cell culture

293T cell lines were obtained from the American Type Cell Collection (Manassas, VA, USA) and were grown in the complete F12 medium (Sigma, St. Louis, MO, USA) with 2.5% fetal bovine serum (FBS), 15% horse serum, 5 μg/mL streptomycin, and 5 U/mL penicillin (Invitrogen, CA, USA). Cells were maintained at 37 ℃ in a humidified atmosphere of 5% CO_2_.

### Quantitative real-time PCR (qRT-PCR)

Total RNA was isolated with Trizol (Invitrogen, MA, USA). Extraction of cytoplasmic and nuclear RNA was performed with a PARISTM Kit (Invitrogen). qRT-PCR reaction was conducted through PrimeScript^TM^ RT Master Mix and SYBR^®^ Premix Ex TaqTM II (Takara, Shiga prefecture, Japan) using the Bio-Rad CFX96 PCR System (Bio-Rad, CA, USA). The primers were synthesized by Invitrogen. Threshold cycle value was calculated with the 2^−ΔΔCt^ method with GAPDH or U6 as a normalization control.

### Cell transfection

CLRN1-AS1 or FOXP1 was overexpressed in PPA cell by transfecting with pcDNA3.1 vectors subcloned with the whole sequence of CLRN1-AS1 or (pcDNA3.1-CLRN1-AS1 or pcDNA3.1-FOXP1). For knockdown of CLRN1-AS1, FOXP1, and DKK1, shRNAs specifically targeting CLRN1-AS1 (sh-CLRN1-AS1#1/2/3), FOXP1 (sh-FOXP1#1/2/3), DKK1 (sh-DKK1#1/2/3), and their negative control shRNAs (sh-NC) were transfected into PPA cell. MiR-217 mimics or inhibitors and negative controls were synthesized and purchased from ThermoFisher Scientific (USA). Transfections were carried out by using Lipofectamine 2000 (Invitrogen, Carlsbad, CA, USA) according to the manufacturer’s instruction.

### Cell counting kit-8 (CCK-8) assay

Cells were seeded in 96-well plates at the density of 2 × 10^3^ cells/well. Cell viability was measured using the CCK-8 assay kit (Dojindo, Japan). Following incubation, 10 μL of CCK-8 solution was added to each well of the 96-well plate and cultured for 2 h in an incubator. After the incubation, phosphate-buffered saline (PBS) was applied to wash the plates twice. The optical density was measured at 450 nm and a proliferation curve based on time and absorbance was generated. Three replications of CCK-8 assay were conducted.

### Colony formation assay

The treated cell lines were planted into six-well plates at 800 cells per well and incubated for 2 weeks. After incubation, cells were subjected to fixation in 4% paraformaldehyde for 15 min and stained in 1 mL of 0.1% crystal violet solution for 30 min. The culture plates were photographed. Visible colonies in each well were quantitated by Image J software. Colony formation assay was in triplicate.

### 5-ethynyl-20-deoxyuridine (EdU) assay

EdU incorporation assay kit was purchased from Ribobio (Invitrogen). The transfected cells were put into 96-well plates and cultured with 100 μL of 50 μM EdU medium diluent for 3 h. Followed by fixation in 4% paraformaldehyde, cells were incubated with 100 μL of the 0.5% Troxin X-100 for 10 min before treatment with 100 μL of 1× Apollo^®^ 488 fluorescent staining solution for 30 min at 37 °C. The nuclei were stained with DAPI. Images were taken using a fluorescent microscope. EdU assay was repeated thrice.

### Caspase-3 activity detection

In accordance with the manufacturers’ recommendation, Caspase-3 activity kit (Abcam, Cambridge, MA, USA) was used to test caspase-3 activity. Briefly, total protein extracted from PPA cells was planted into 96-well plates, followed by incubation with reaction buffer and caspase-3 substrate at 37 °C for 4 h. Caspase-3 activity was assessed using a microplate reader (Tecan Group Ltd., Männerdorf, Switzerland) at 405 nm. This experiment was carried out in triplicate.

### JC-1 assay

PPA cells were placed into 96-well plates with a density of 10,000 cells each well and cultured all night. After treatment with Cis and/or mdivi-1, with or without 2 mM NAC or 0.5 mM TEMPOL for 18 h, this assay was conducted using cationic, lipophilic dye qo-1,1′,3,3′ tetraethylbenzimidazolyl carbocyanine iodide (JC-1, Cayman Chemical, Ann Arbor, MI, USA) following the user guide. Subsequently, the centrifuged cells were loaded in JC-1 for half an hour and cultured in assay buffer. ΔΨ_m_ was evaluated by a fluorescent plate reader, Gemini XPS and images were acquired by fluorescence microscope. The final result was acquired from three different biological replications.

### In vivo assay

Six-week-old male BALB/C athymic nude mice were acquired commercially from the National Laboratory Animal Center (Beijing, China) and kept under specific pathogen-free conditions. The animal experiment was approved by the Animal Research Ethics Committee of The Affiliated Cancer Hospital of Harbin Medical University. Cell lines transfected with pcDNA-CLRN1-AS1 or empty vector were reaped and subcutaneously injected into the left flank of the nude mice (five mice per group). Tumor volume was examined every fourth day and measured as length × width^2^ × 0.5. Four weeks after injection, mice were killed. Tumor tissues were excised and weighed for further analysis.

### Ago2-RNA immunoprecipitation (RIP) assay

PPA cells grown to 70–80% confluence were rinsed in precold PBS and lysed in RIP buffer containing 0.5% Nonidet, 0.5 mM DTT, 20 mM Tris-HCL pH7.5, 150 mM KCL, 2 mM EDTA, 1 mM NaF, and inhibitors of 1 RNases, proteases, and phosphatases. Thereafter, cell lysate was co-immunoprecipitated with anti-CLRN1-AS1 (Abcam), anti-Ago2 (Sigma-Aldrich, MO, USA), or anti-IgG (Abcam) antibodies bound to sepharose beads at 4 °C all night. At last, the retrieved CLRN1-AS1 was purified and subjected to qRT-PCR analysis to demonstrate its presence in RISC complex. Total RNA was seen as the input control. Triplicated experiments were conducted.

### Microarray analysis

After quantile normalization, raw signals obtained from microarrays were log2 transformed. The absolute value of fold change (FC) > 1.5 and the *P*-value < 0.05 indicated that lncRNAs were differentially expressed in pituitary prolactinoma samples.

### Western blot assay

Protein specimens were diluted in loading buffer and denatured at 95 °C. After electrophoresing on 10% sodium dodecyl sulfate polyacrylamide gel electrophoresis (SDS-PAGE), total protein (40 μg) was transferred onto the polyvinylidene difluoride membrane. Five percent skimmed milk was used to block the membrane at room temperature for 2 h. Primary antibodies including anti-β-catenin, anti-c-myc, anti-cyclin D1, anti-p62, anti-Beclin1, anti-DKK1, and anti-GAPDH, were purchased from Abcam (USA). The antibody against LC3I/II (#ABC929) was bought from Sigma-Aldrich (Saint-Louis, MO, USA). The membrane was incubated with the indicated antibodies overnight at 4^o^C, followed by washing and incubation with corresponding secondary antibodies (Abcam) for 2 h. At length, protein samples were subjected to an enhanced chemiluminescence detection system (Bio-Rad lab, Hercules, CA, USA). This assay was performed more than three times.

### Subcellular fractionation assay

According to recommendation provided by supplier, subcellular fractionation assay was carried out by using PARIS™ Kit (Invitrogen). PPA cells in cell fractionation buffer were subjected to centrifugation. The supernatant was collected. The remaining lysates were rinsed in cell fractionation buffer. Cell disruption buffer was utilized to lyse cell nuclei. Thereafter, the lysate was cultured with the supernatant, 2× lysis/binding solution, and ethanol. At last, qRT-PCR was used to determine the cytoplasmic and nuclear RNAs. Each procedure was repeated thrice.

### Fluorescence in situ hybridization (FISH)

CLRN1-AS1-FISH probe was designed and synthesized by Invitrogen. PPA cells were placed on culture slides and fixed in 4% paraformaldehyde, following sealing with prehybridization buffer at 37 °C for 4 h. FISH probe was added into the hybridization mixture all night. Afterward, slides were washed in washing buffer containing saline-sodium citrate. Cells were stained with DAPI for visualizing nuclei. Lastly, cells were observed under an Olympus fluorescence microscope (IX73; Olympus Corp., Tokyo, Japan). Experiments were performed for three times for biological replicates.

### DNA pull-down assay

Tweenty micrograms of biotinylated CLRN1-AS1 promoter or no-biotinylated CLRN1-AS1 promoter probes were treated with streptavidin-coated Dynabeads (Invitrogen) for half an hour in binding buffer containing 5 mM Tris-base, 0.5 mM EDTA, 1 M NaCl, and 0.003% NP-40. Afterward, oligonucleotide-bead complex was cultured with 100 μg of nuclear protein at room temperature for 2 h. Followed by washing in PBS, protein was subjected to elution through boiling in 1× sample buffer containing 50 mM Tris, pH 6.8, 10% glycerol, 5% 2-mercaptoethanol, 2% SDS, and 0.2% bromophenol blue. Experimental procedures were conducted for at least three times.

### Chromatin immunoprecipitation (ChIP)

PPA cells were fixed in 1% formaldehyde for 10 min at room temperature. The cross-linking was terminated with the addition of 125 mM glycine. The cells were washed twice by cold PBS, then resolved in a buffer containing 1 mM phenylmethanesulfonyl fluoride, 10 mM Tris-HCl (pH 8.0), 1% Triton X-100 and protease inhibitor cocktail, and 1% sodium deoxycholate for 10 min at 4 ℃. Sonication was performed for shearing chromatin into 500-bp fragments with a Bioruptor^®^ Sonicator (Diagenode s.a., Seraing, Belgium). The supernatant was collected after centrifugation (16,000 × g for 100 min at 4 ℃) and equally divided into six tubes (100 μl/tube). The suitable antibodies containing anti-FOXP1 or anti-IgG obtained from Abcam were added into each tube and incubated at 4 ℃ for 3 h. ChIP-grade agarose beads linked with protein FOXP1 was used for Immunoprecipitation. Finally, the immunoprecipitated RNAs were quantified by qRT-PCR.

### Luciferase reporter assay

The wild type sequence of CLRN1-AS1 or DKK1 3’ UTR containing the binding sites with miR-217 (CLRN1-AS1-WT or DKK1-WT) was cloned into the firefly luciferase gene in pmirGLO luciferase vector (Promega, Madison, WI, USA). The mutant form of CLRN1-AS1-AS1 or DKK1 (CLRN1-AS1-AS1-MUT or DKK1-MUT) was established using the GeneTailor™ Site-Directed Mutagenesis System (Invitrogen). PPA cells in 24-well plates were separately cotransfected with the aforementioned reporter plasmids and miR-217 mimics or miR-NC, as well as miR-217 inhibitors or anti-miR-NC using Lipofectamine2000.

For CLRN1-AS1 promoter luciferase analysis, CLRN1-AS1 promoter containing FOXP1 binding sites was cloned into pGL3-Basic reporter vector (Promega). Cells were cotransfected with indicated reporter plasmids and sh-FOXP1, pcDNA-FOXP1, or negative controls. At last, luciferase activities were evaluated by Dual-Luciferase Reporter Assay System (Promega Corporation, Fitchburg, WI, USA), normalizing to Renilla luciferase activity. All results were acquired from triplicated independent experiments.

### Bioinformatics analysis

MiRNAs containing the binding sites with CLRN1-AS1 were predicted using starBase v3.0 (http://starbase.sysu.edu.cn/). DNA motif of FOXP1 and putative binding sequences of FOXP1 in CLRN1-AS1 promoter were obtained from JASPAR (http://jaspar.genereg.net/).

### Statistical analysis

Data were exhibited as mean ± standard deviation from triplicated independent experiments. SPSS 18.0 software (SPSS Inc., Chicago, IL, USA) was used for statistical analyses. The difference of two independent groups was analyzed by a two-tailed Student’s *t* test, while multi-group comparison was made by ANOVA. Expression correlation between genes was analyzed by Pearson correlation analysis. *P*-values < 0.05 were considered statistically significant.

## Results

### Downregulation of CLRN1-AS1 inactivated Wnt/β-catenin signaling pathway in pituitary prolactinoma

**Fig. 1:**
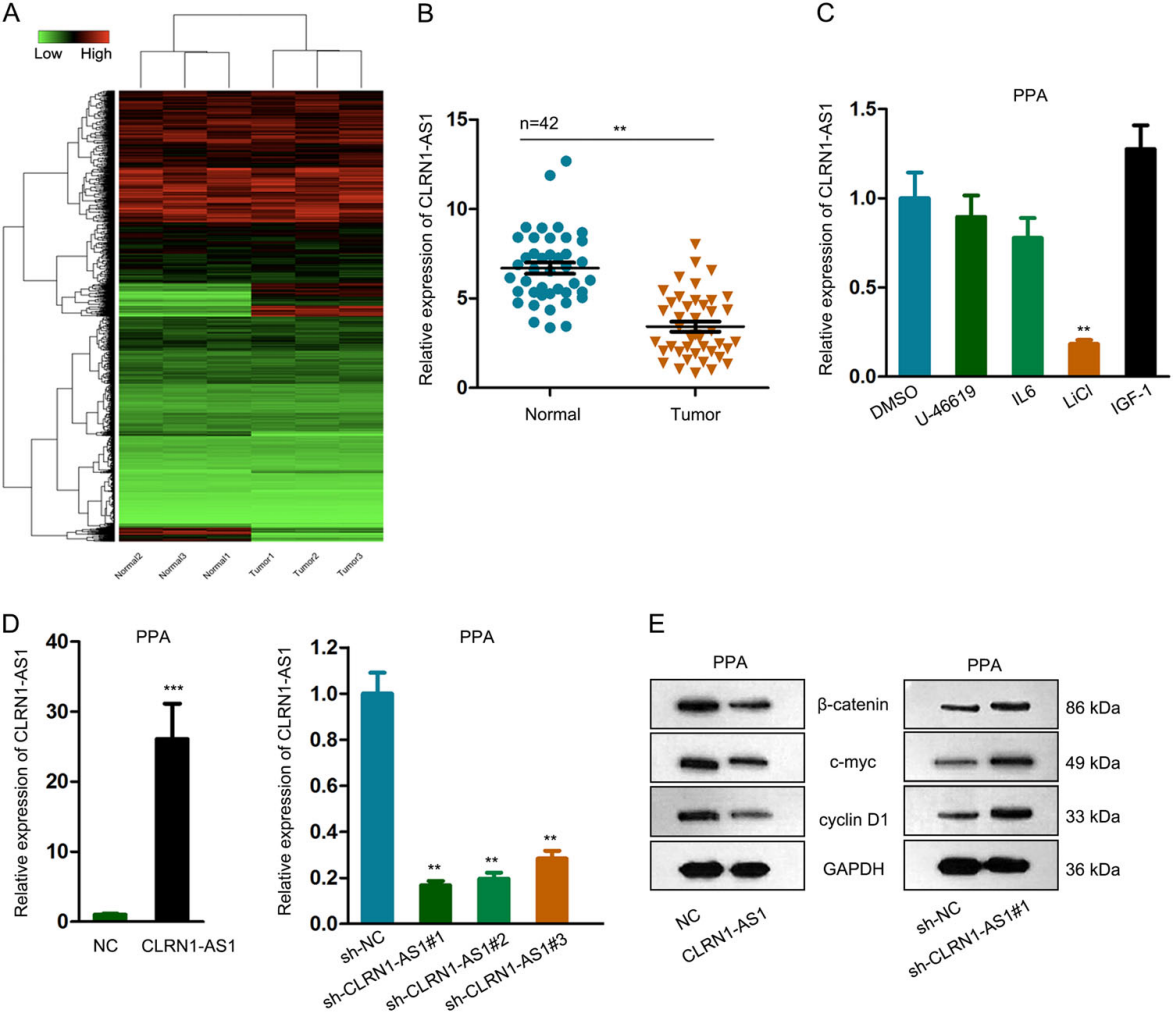
Downregulation of CLRN1-AS1 inactivated Wnt/β-catenin signaling pathway in pituitary prolactinoma. **a** Microarray analysis of differentially expressed lncRNAs in pituitary prolactinoma samples. **b** Expression level of CLRN1-AS1 in 42 pituitary prolactinoma samples compared to adjacent normal samples. **c** The expression level of CLRN1-AS1 was examined in cells treated with activators of several classical signaling pathways. Cells treated with DMSO was used as negative control. **d** Overexpression and knockdown efficiency for CLRN1-AS1 in PPA cells. **e** The protein levels of Wnt/β-catenin signaling pathway factors (β-catenin, c-myc, and cyclin D1) in CLRN1-AS1-downregulated or upregulated cells. ^**^P < 0.01, ^***^P < 0.001 versus control group, indicated data are statistically significant

At first, we applied microarray analysis to find out lncRNAs that were differentially expressed in pituitary prolactinoma samples. As presented in Fig. 1a, there were 193 lncRNAs that were significantly downregulated in pituitary prolactinoma samples in addition to 273 upregulated lncRNAs. Among all downregulated lncR NAs, CLRN1-AS1 presented highest FC, thus we chose it for further analysis. Low expression level of CLRN1-AS1 was further identified in 42 pituitary prolactinoma samples compared to adjacent normal samples (Fig. 1b). It has been widely reported that lncRNAs may exert functions in malignant tumors by cooperating with signaling pathways. To explore whether CLRN1-AS1 regulated a certain signaling pathway in pituitary prolactinoma, we treated PPA cells with the activators of several classical signaling pathways to observe the expression change of CLRN1-AS1. It was found that CLRN1-AS1 expression was obviously downregulated by supplementing with LiCl (the activator of the Wnt/β-catenin signaling pathway) (Fig. 1c). Next, we separately overexpressed or silenced CLRN1-AS1 in PPA cells. The overexpression and knockdown efficiency for CLRN1-AS1 was identified and shown in Fig. 1d. The sh-CLRN1-AS1#1 exhibited highest knockdown efficiency, thus we chose it for subsequent experiments. For further confirmation, we examined the protein levels of Wnt/β-catenin signaling pathway factors. The results revealed that protein levels of β-catenin, c-myc, and cyclin D1 were negatively regulated by CLRN1-AS1 (Fig. 1e), indicating that CLRN1-AS1 potentially inactivated the Wnt/β-catenin signaling pathway in pituitary prolactinoma.

### CLRN1-AS1 suppressed pituitary prolactinoma cell growth

**Fig. 2:**
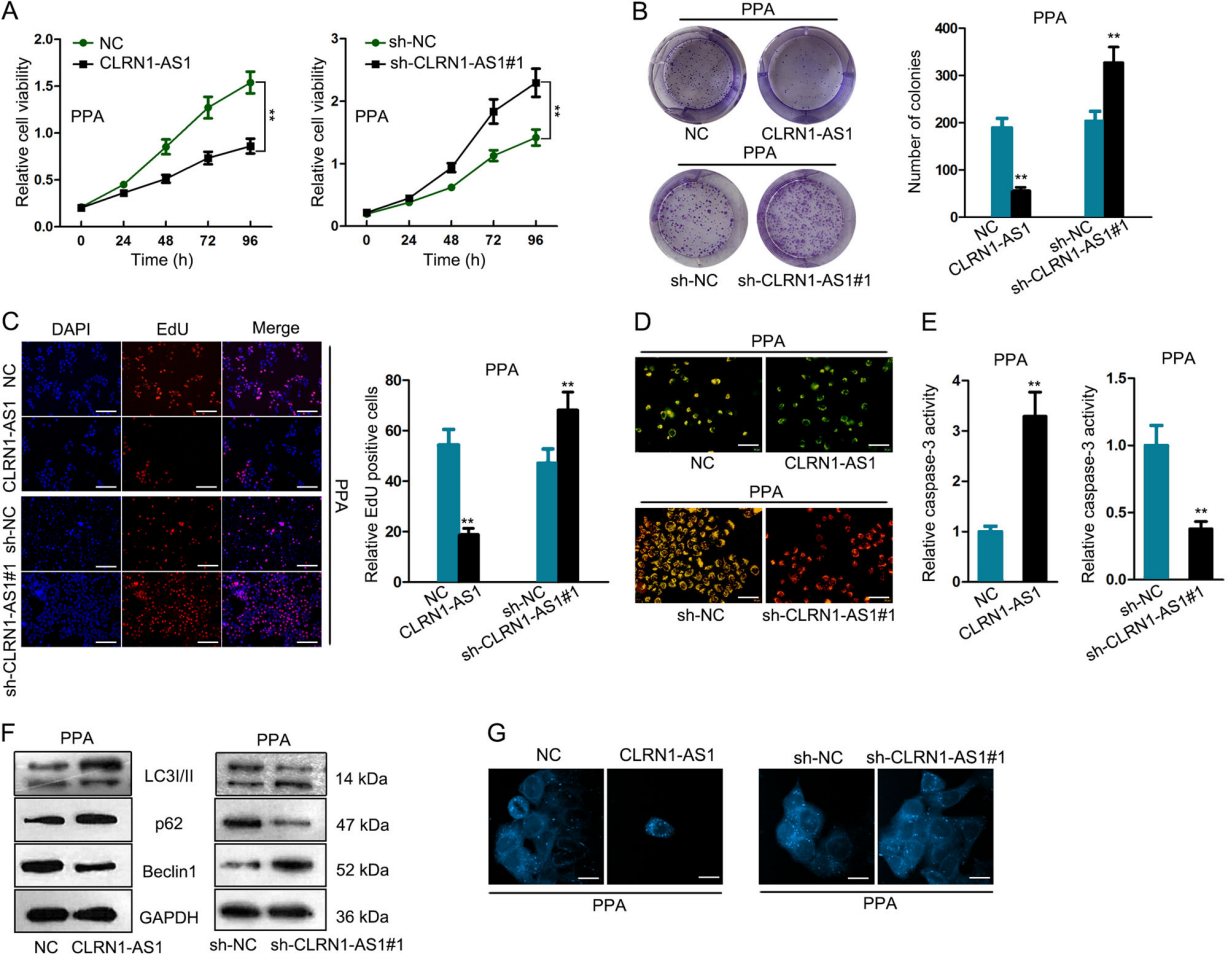
CLRN1-AS1 suppressed pituitary prolactinoma cell growth. **a**–**c** Cell proliferation assays were conducted to determine the effect of CLRN1-AS1 overexpression or knockdown on cell proliferation. **d**–**e** JC-1 assay and caspase-3 activity test were separately performed to demonstrate the role of CLRN1-AS1 in regulating cell apoptosis. **f** The levels of autophagy-related proteins in indicated pituitary prolactinoma cell lines. **g** The ratio of autophagosome in two transfected PPA cells. ^**^P < 0.01 versus control group, indicated data are statistically significant

To identify the function of CLRN1-AS1 in regulating cellular processes, we conducted loss or gain of function assays in indicated PPA cells. Analysis of cell proliferation revealed that overexpression of CLRN1-AS1 efficiently inhibited cell proliferation, while silencing of it promoted cell proliferation (Fig. 2a–c). Moreover, cell apoptosis condition was observed in indicated cells. After JC-1 assay and caspase-3 activity test, CLRN1-AS1 expression was identified to be positively correlated with cell apoptosis (Fig. 2d, e). Autophagy is a crucial biological process that is closely associated with cell death. To investigate the role of CLRN1-AS1 in autophagy, we tested the level of autophagy-related proteins and the ratio of autophagosome in two transfected cells (Fig. 2f, g). According to the experimental results, we found that CLRN1-AS1 induced inhibition of autophagy in pituitary prolactinoma cell lines.

### CLRN1-AS1 acted as a sponge of miR-217 in pituitary prolactinoma cell lines

**Fig. 3:**
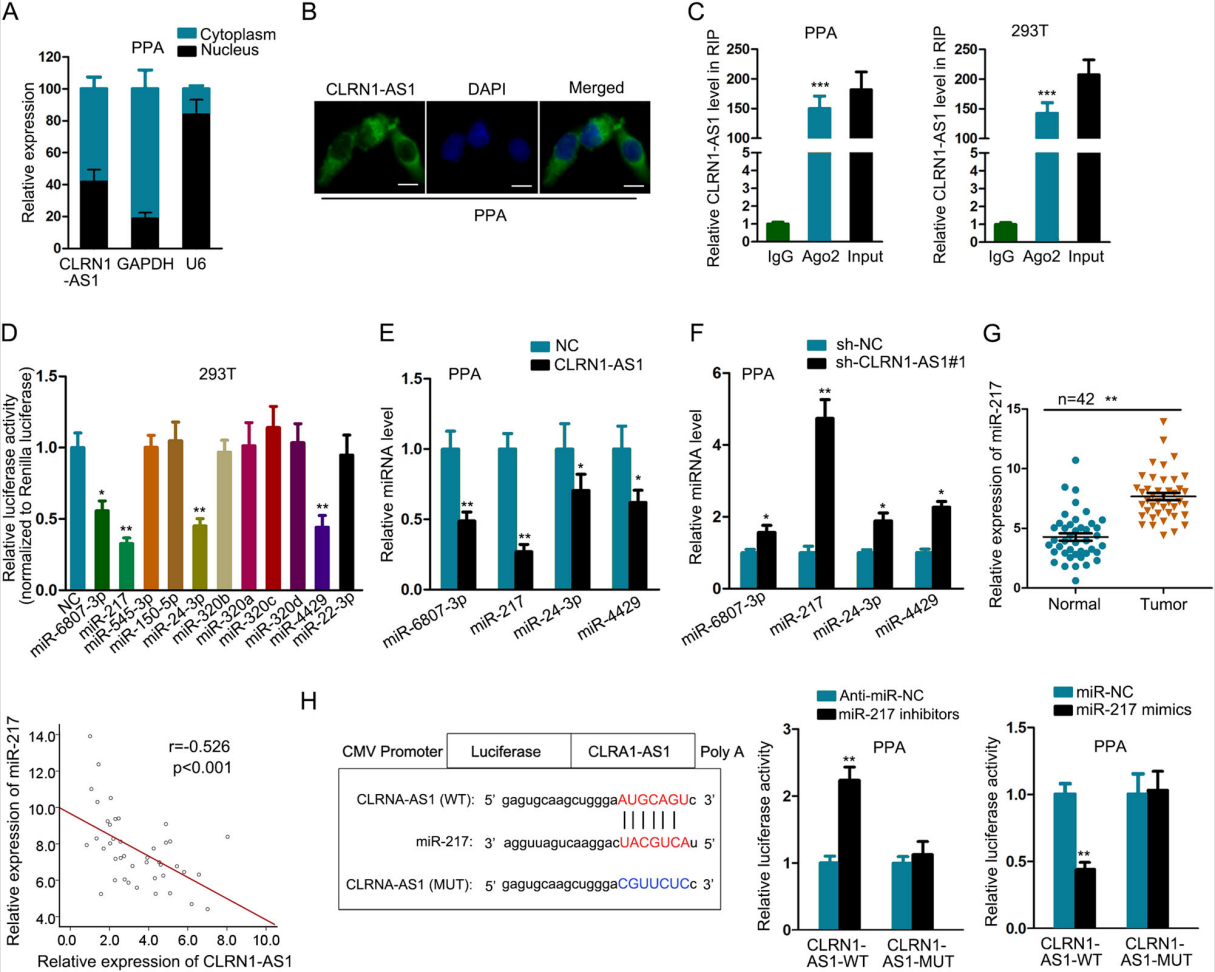
CLRN1-AS1 acted as a sponge of miR-217 in pituitary prolactinoma cell lines. **a**, **b** Localization of CLRN1-AS1 in pituitary prolactinoma cell lines. **c** The binding of CLRN1-AS1 to RISC was detected by Ago2-RIP assay. **d** Eleven miRNAs that potentially bind with CLRN1-AS1 were subjected to luciferase reporter assay. **e**, **f** Expression level of four miRNAs in response to the overexpression or knockdown of CLRN1-AS1. **g** miR-217 expression in pituitary prolactinoma samples and the expression correlation between miR-217 and CLRN1-AS1. **h** The binding sequence between CLRN1-AS1-WT/MUT and miR-217 was used for luciferase reporter assay. ^*^P < 0.05, ^**^P < 0.01, ^***^P < 0.001 versus control group, indicated data are statistically significant

Mechanistically, lncRNAs can exert functions by transcriptionally or posttranscriptionally regulating gene expression. To detect the potential molecular mechanism of CLRN1-AS1, we identified the cellular localization of it in pituitary prolactinoma cell lines. According to the data obtained from subcellular fractionation assay and FISH, CLRN1-AS1 tended to be located in the cytoplasm of pituitary prolactinoma cell lines (Fig. 3a, b). LncRNAs can posttranscriptionally regulate gene expression by acting as ceRNAs. In this regard, we applied Ago2-RIP to identify the ceRNA potential of CLRN1-AS1. The results showed that CLRN1-AS1 could bind with RISC, suggesting it might exert ceRNA function (Fig. 3c). Searching from starBase, there are 11 miRNAs that potentially bind with CLRN1-AS1. To determine which miRNA can actually interact with CLRN1-AS1, we detected the luciferase activity of reporter vector containing the sequence of CLRN1-AS1 by cotransfecting with these 11 miRNAs. As a result, the luciferase activity was efficiently decreased by miR-6807-3p, miR-217, miR-24-3p, and miR-4429 (Fig. 3d). Then, we examined the expression level of these four miRNAs in response to the overexpression or knockdown of CLRN1-AS1. The results suggested that miR-217 was most efficiently regulated by CLRN1-AS1 (Fig. 3e, f). Therefore, we further examined the expression level of it in paired tissue samples. As expected, miR-217 was expressed at a high level in pituitary prolactinoma samples, which exhibited a negative correlation with the expression of CLRN1-AS1 (Fig. 3g). For next experiments, miR-217 expression was increased or decreased by transfecting with miR-217 mimics or inhibitors (Supplementary Fig. 1a). Subsequently, the binding sequence between CLRN1-AS1 and miR-217 was predicted and listed. Both wild and mutant sequences were cloned into luciferase reporter vectors and subjected to luciferase activity analysis. Unsurprisingly, cotransfection with miR-217 mimics or inhibitors obviously affected the luciferase activity of CLRN1-AS1-WT vector but not CLRN1-AS1-MUT (Fig. 3h). Finally, Ago2-RIP assay demonstrated that CLRN1-AS1 and miR-217 were both pulled down by Ago2 antibody (Supplementary Fig. 2a).

### MiR-217 mediated the function of CLRN1-AS1

**Fig. 4:**
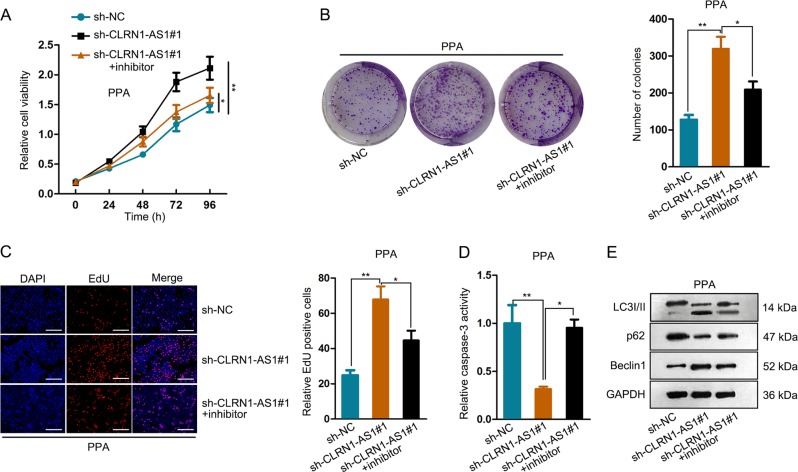
MiR-217 mediated the function of CLRN1-AS1. **a**–**c** CCK-8, colony formation and EdU assays revealed the effect of miR-217 inhibitors on sh-CLRN1-AS1-induced cell proliferation. **d** Caspase-3 activity test revealed that cell apoptosis suppressed by the knockdown of CLRN1-AS1. **e** Similarly, we found that autophagy promoted by the inhibition of CLRN1-AS1 was recovered by the cotransfection of miR-217 inhibitor. ^*^P < 0.05, ^**^P < 0.01 versus control group, indicated data are statistically significant

To demonstrate the involvement of miR-217 in CLRN1-AS1-mediated function, rescue assays were carried out in PPA cells. According to the result of CCK-8, colony formation, and EdU assays, inhibition of miR-217 expression reversed the positive effect of sh-CLRN1-AS1#1 on cell proliferation (Fig. 4a–c). In addition, caspase-3 activity test revealed that cell apoptosis suppressed by the knockdown of CLRN1-AS1 was promoted by cotransfecting with miR-217 inhibitor (Fig. 4d). Similarly, we found that autophagy promoted by the inhibition of CLRN1-AS1 was recovered by the cotransfection of miR-217 inhibitor (Fig. 4e). Combining with the above findings, we confirmed that CLRN1-AS1 regulated pituitary prolactinoma cell survival by sponging miR-217.

### DKK1 competed with CLRN1-AS1 for binding with miR-217

**Fig. 5:**
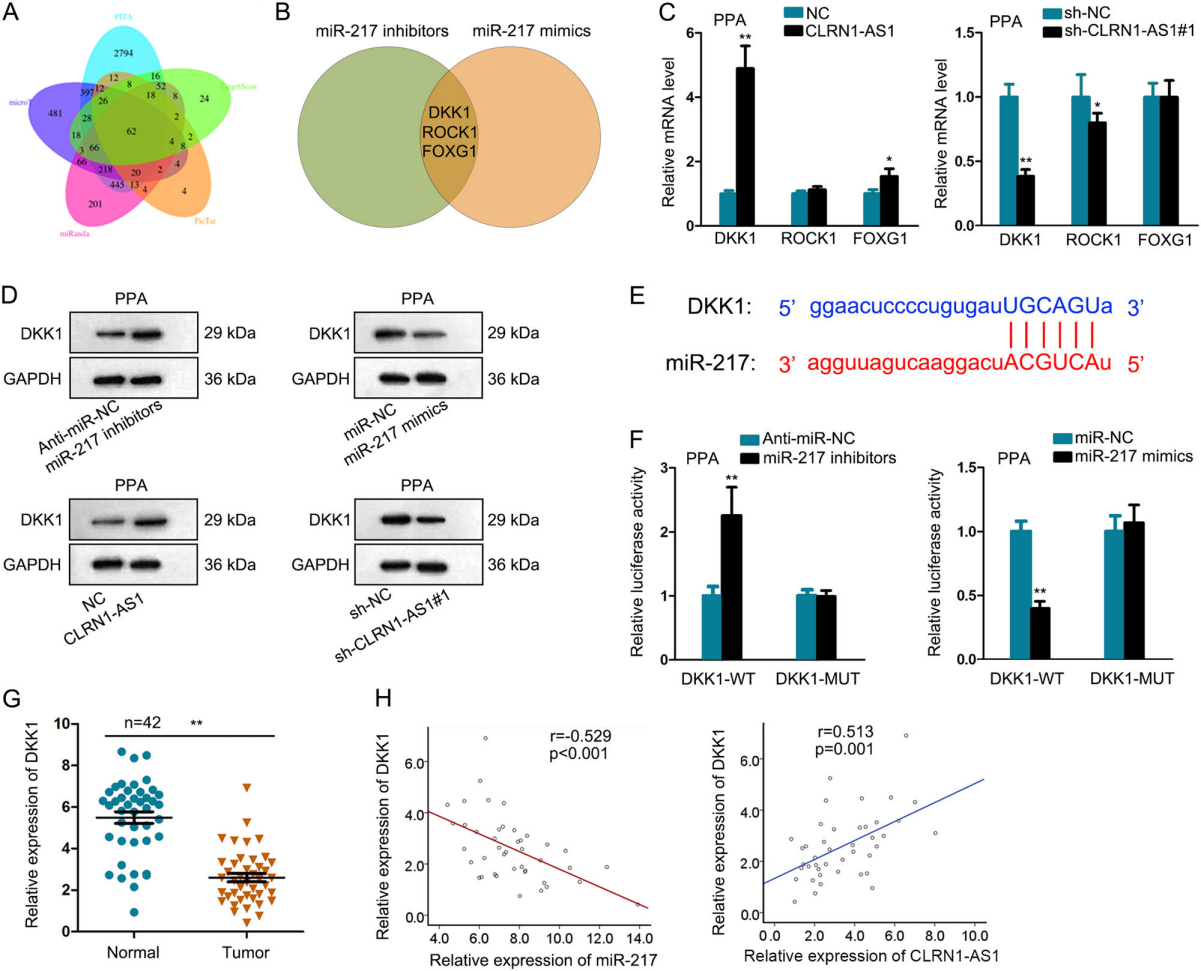
DKK1 competed with CLRN1-AS1 for binding with miR-217. **a** 62 putative targets of miR-217 was predicted by using five bioinformatics prediction tools. **b** Expression level of these 62 mRNAs in cell transfecting with miR-217 mimics or inhibitor. **c** mRNA levels of three potential targets in cells transfected with sh**-**CLRN1-AS1 or pcDNA-CLRN1-AS1 expression vector. **d** Protein level of DKK1 was tested in cells in response to the differential expression of miR-217 or CLRN1-AS1. **e** The putative binding sequence between miR-217 and DKK1 was listed. **f** Luciferase activity analysis revealed that miR-217 mimics or inhibitors significantly regulated the luciferase activity of DKK1-WT but not DKK1-MUT. **g** Relative expression of DKK1 in pituitary prolactinoma samples. **h** Expression association between miR-217 and DKK1 as well as between CLRN1-AS1 and DKK1 in tumor samples. ^*^P < 0.05, ^**^P < 0.01 versus control group, indicated data are statistically significant

In this step, we detected the potential target mRNAs of miR-217 using five bioinformatics prediction tools. As a result, there are 62 putative targets of miR-217 (Fig. 5a). Then, we examined the expression level of these 62 mRNAs in cell transfecting with miR-217 mimics or inhibitor. Three of them were significantly negatively regulated by miR-217 (Fig. 5b). Among these three mRNAs, DKK1 was significantly positively regulated by CLRN1-AS1 (Fig. 5c). The protein level of DKK1 was also tested in cells in response to the differential expression of miR-217 or CLRN1-AS1 (Fig. 5d). The protein levels exhibited a consistent tendency with mRNA level. Then, the putative binding sequence between miR-217 and DKK1 was listed (Fig. 5e). Further luciferase activity analysis revealed that miR-217 mimics or inhibitors significantly regulated the luciferase activity of DKK1-WT but not DKK1-MUT (Fig. 5f). Moreover, both miR-217 and DKK1 were enriched in Ago2 containing beads (Supplementary Fig. 2b). Subsequently, we determined the relative low expression level of DKK1 in pituitary prolactinoma samples (Fig. 5g). The expression association between miR-217 and DKK1 was shown to be negative, while the expression correlation between CLRN1-AS1 and DKK1 in tumor samples was found to be positive (Fig. 5h). These data indicated that CLRN1-AS1 upregulated DKK1 by sponging miR-217.

### DKK1/Wnt/β-catenin signaling pathway involved in CLRN1-AS1-mediated pituitary prolactinoma cell survival

**Fig. 6:**
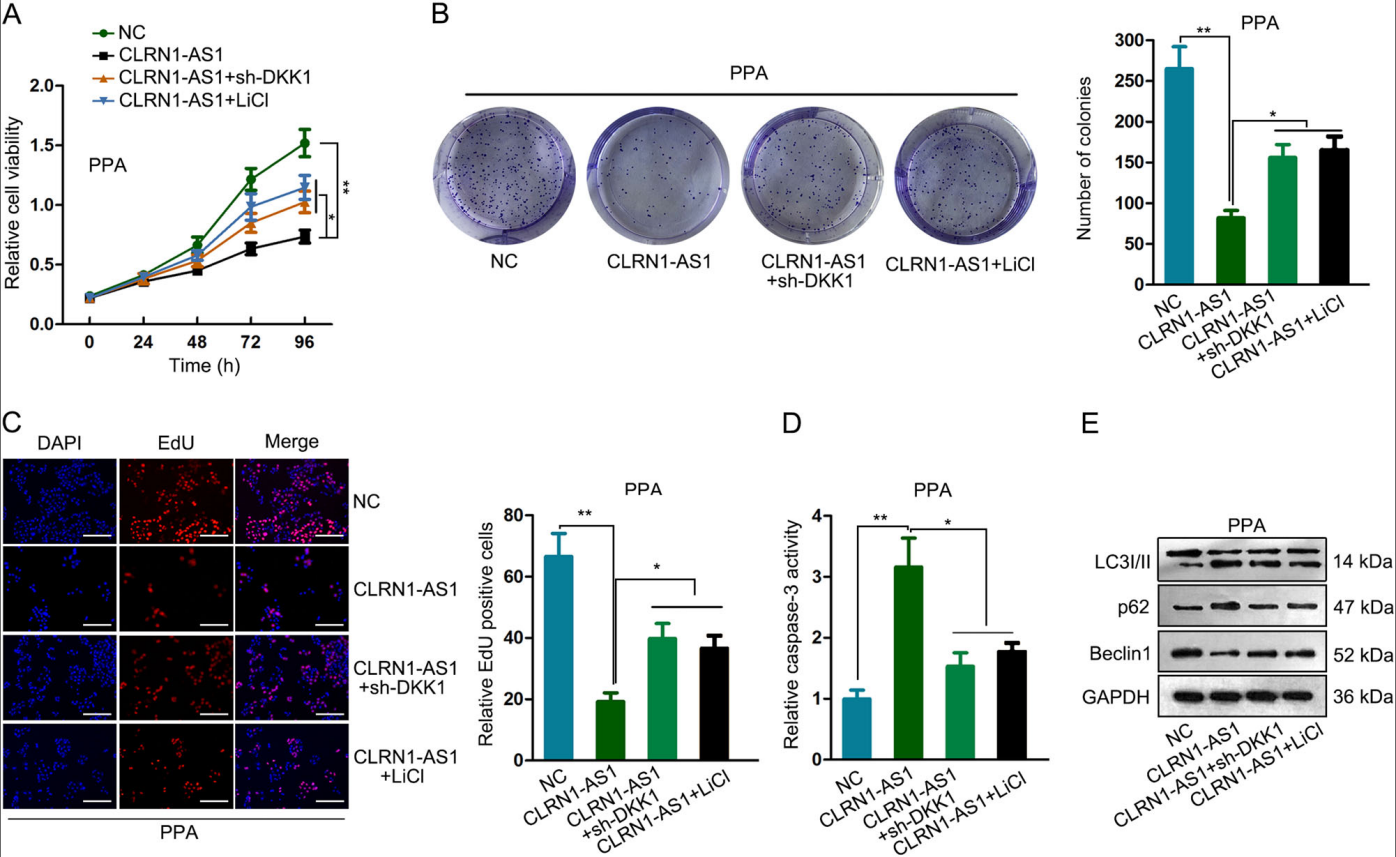
DKK1/Wnt/β-catenin signaling pathway involved in CLRN1-AS1-mediated pituitary prolactinoma cell survival. **a**–**c** The inhibitory effect of CLRN1-AS1 overexpression on cell proliferation was reversed by introducing sh-DKK1 or LiCl. **d**, **e** Treatment of LiCl or sh-DKK1 reversed the effect of CLRN1-AS1 overexpression on apoptosis and autophagy. ^*^P < 0.05, ^**^P < 0.01 versus control group, indicated data are statistically significant

According to our previous experimental data, CLRN1-AS1 might inactivate the Wnt/β-catenin signaling pathway. DKK1 is an inhibitor of the Wnt/β-catenin signaling pathway. Combining with all these findings, we proposed that CLRN1-AS1 upregulated DKK1 to inactivate the Wnt/β-catenin signaling pathway. To validate the role of the DKK1 and Wnt/β-catenin signaling pathway in CLRN1-AS1-mediated functions, we conducted rescue assays in PPA cell by applying DKK1-specific shRNA (sh-DKK1) and LiCl. Knockdown efficiency for DKK1 was shown in Supplementary Fig. 1b. Interestingly, the inhibitory effect of CLRN1-AS1 overexpression on cell proliferation was reversed by introducing sh-DKK1 or LiCl (Fig. 6a–c). In addition, treatment of LiCl or sh-DKK1 reversed the effect of CLRN1-AS1 overexpression on apoptosis and autophagy (Fig. 6d, e). Based on all these data, we concluded that CLRN1-AS1 suppressed pituitary prolactinoma cell survival by sponging miR-217 to upregulate DKK1 and inactivate the Wnt/β-catenin signaling pathway. In vivo experiments were conducted to further determine the role of CLRN1-AS1 in cell growth. It was found that tumor growth was inhibited after overexpression of CLRN1-AS1 (Supplementary Fig. 3a–c). The expression level of CLRN1-AS1 and DKK1 was increased after CLRN1-AS1 overexpression (Supplementary Fig. 3d).

### FOXP1 acted as a transcription suppressor for CLRN1-AS1

**Fig. 7:**
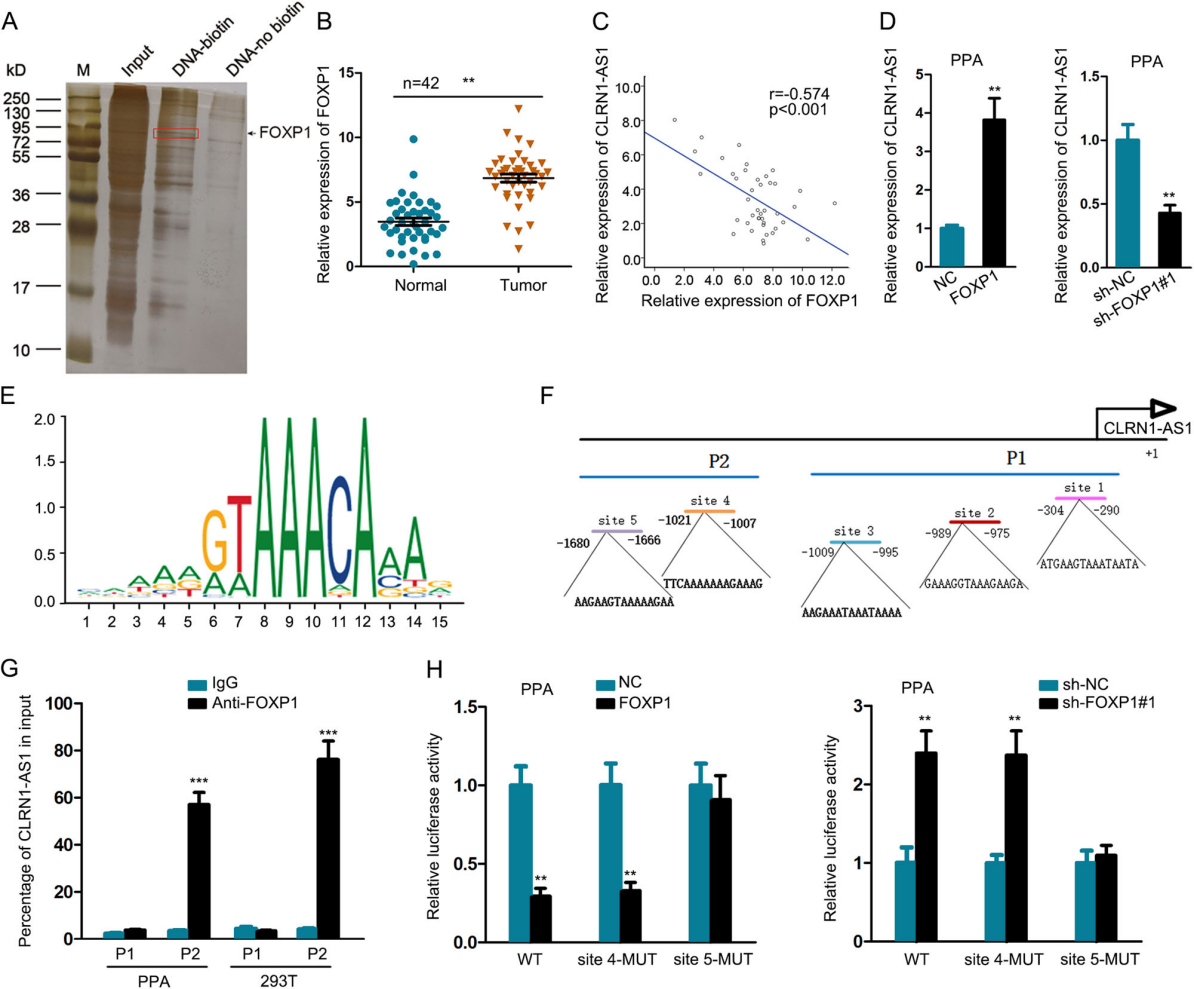
FOXP1 acted as a transcription suppressor for CLRN1-AS1. **a** DNA-pull down assay revealed the interaction between FOXP1 transcription suppressor and CLRN1-AS1. **b** The expression level of FOXP1 was in tumor samples and normal samples. **c** Correlation analysis of expression correlation between FOXP1 and CLRN1-AS1 in tumor samples. **d** qRT-PCR analysis of CLRN1-AS1 expression in PPA cells transfected with sh-FOXP1 or pcDNA-FOXP1. **e**, **f** DNA motif of FOXP1 and predicted five binding sites of FOXP1 in CLRN1-AS1 promoter. **g** ChIP assay revealed that P2 was responsible for the affinity of FOXP1 to CLRN1-AS1 promoter. **h** The effect of FOXP1 overexpression or knockdown on luciferase activity of vector containing CLRN1-AS1 promoter was abolished when site 5 was mutated. ^**^P < 0.01, ^***^P < 0.001 versus control group, indicated data are statistically significant

**Fig. 8:**
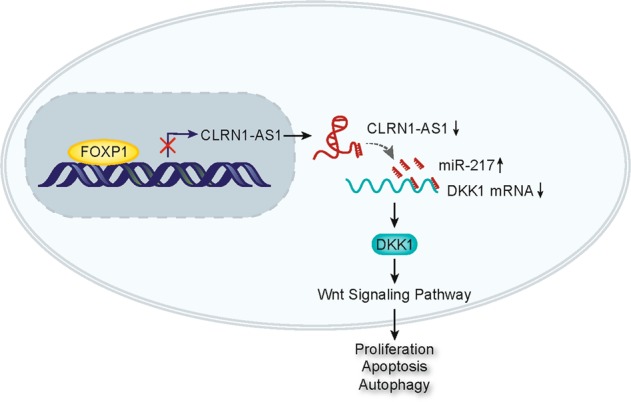
FOXP1-induced CLRN1-AS1 suppressed pituitary prolactinoma by acting as a ceRNA to regulate DKK1/Wnt/β-catenin pathway

In order to analyze the upstream molecular mechanism of CLRN1-AS1, we performed DNA-pull down assay. As presented in Fig. 7a, CLRN1-AS1 was pulled down by FOXP1, which has been demonstrated to be a transcription suppressor^21^. The expression level of FOXP1 was higher in tumor samples than normal samples (Fig. 7b). Correlation analysis showed that there was a negative expression correlation between FOXP1 and CLRN1-AS1 in tumor samples (Fig. 7c). To identify the regulatory effect of FOXP1 on CLRN1-AS1 expression, we separately overexpressed and silenced FOXP1 in PPA cells (Supplementary Fig. 1c). Through qRT-PCR analysis, we determined that FOXP1 negatively regulated CLRN1-AS1 expression in PPA cells (Fig. 7d). Next, we obtained the DNA motif of FOXP1 and predicted five binding sites of FOXP1 in CLRN1-AS1 promoter (Fig. 7e, f). Five binding sequences were divided into part 1 (P1) and part 2 (P2) of CLRN1-AS1 promoter. ChIP assay revealed that P2 was responsible for the affinity of FOXP1 to CLRN1-AS1 promoter (Fig. 7g). Accordingly, we hypothesized that site 4 and site 5 might be responsible for the binding between FOXP1 and CLRN1-AS1 promoter. Then, we constructed luciferase reporter vector containing CLRN1-AS1 promoter or vector containing mutant site 4 or site 5. The results showed that the luciferase activity of vectors containing wild sequences was decreased or increased by overexpression or knockdown of FOXP1. However, the effect of FOXP1 overexpression or knockdown was abolished when site 5 was mutated (Fig. 7h). Therefore, site 5 was responsible for the transcription inhibition of FOXP1 on CLRN1-AS1. All these findings suggested that FOXP1-induced CLRN1-AS1 suppressed cell growth in pituitary prolactinoma by acting as a ceRNA to regulate the DKK1/Wnt/β-catenin signaling pathway (Fig. 8).

## Discussion

Pituitary prolactinoma is the commonest subtype of pituitary adenoma^3^. A large proportion of prolactinoma is benign but may result in significant morbidity correlated to excessive hormone production, as well as symptoms of mass effect^22^. The biological processes of pituitary prolactinoma are complicated. In recent years, the function and mechanism of lncRNAs have been studied and reported. However, the role of lncRNAs in pituitary prolactinoma is still little studied. Therefore, it is necessary to reveal the functional mechanism of lncRNAs in the tumorigenesis and progression of pituitary prolactinoma. In our present study, we investigated an lncRNA that was significantly downregulated in pituitary prolactinoma samples through microarray analysis. Moreover, lncRNA CLRN1-AS1 was downregulated in 42 tumor samples compared to normal samples.

IL6/STAT3, Wnt/β-catenin, p38/MAPK, and PI3K/AKT signaling pathways have been reported to be crucial mediators in the progression of malignant tumors^23–26^. In our present study, we found that the expression level of CLRN1-AS1 was decreased a lot in PPA cells treated with LiCl, indicating the potential association between the CLRN1-AS1 and Wnt/β-catenin signaling pathway. Then, we investigated the regulatory effect of CLRN1-AS1 on the proteins of the Wnt/β-catenin signaling pathway. According to the results, we concluded that CLRN1-AS1 was downregulated in pituitary prolactinoma and inactivated the Wnt/β-catenin signaling pathway. Functionally, dysregulation of lncRNAs can affect various biological processes in tumor progression^27,28^. Accordingly, we performed loss or gain of function assays to determine the role of CLRN1-AS1 in cellular processes of pituitary prolactinoma. Interestingly, CLRN1-AS1 was found to be able to suppress cell proliferation and promote apoptosis. Autophagy is a crucial biological behavior when cell survival was changed. In our current study, we discovered that CLRN1-AS1 partly suppressed autophagy of pituitary prolactinoma cells. Therefore, we identified the anti-oncogenic property of CLRN1-AS1 in pituitary prolactinoma.

Cytoplasmic localization of CLRN1-AS1 prompted us to determine its posttranscriptional regulatory mechanism in pituitary prolactinoma. As we all know, ceRNA is a common mechanism of lncRNAs, among which lncRNA upregulate a certain mRNA by sponging a miRNA^29,30^. After bioinformatics analysis and mechanism investigation, we determined that CLRN1-AS1 acted as a molecular sponge of miR-217 in pituitary prolactinoma. Functionally, the effect of CLRN1-AS1 on pituitary prolactinoma cell growth was affected by miR-217. Therefore, we confirmed that CLRN1-AS1 suppressed pituitary prolactinoma cell growth by sponging miR-217. Similarly, the target mRNA of miR-217 was predicted. Among all candidates, DKK1 was simultaneously regulated by CLRN1-AS1 and miR-217. More importantly, DKK1 is known as an inhibitor of the Wnt/β-catenin signaling pathway. Combining with our previous experimental results, we speculated that CLRN1-AS1 acted as a ceRNA to upregulate DKK1, thereby inactivating the Wnt/β-catenin signaling pathway in pituitary prolactinoma. Rescue assays demonstrated the involvement of the DKK1 and Wnt/β-catenin signaling pathway in CLRN1-AS1-mediated pituitary prolactinoma cell growth.

CLRN1-AS1 was downregulated in pituitary prolactinoma and exerted tumor-suppressive functions. Therefore, it is significant to explore its upstream molecular mechanism. Through DNA pull down and mass spectrometry analysis, we determined that FOXP1 is a potential transcription regulator for CLRN1-AS1. Based on previous studies, upregulation of FOXP1, which is activated by transcription promoter, can promote tumorigenesis^31^. In this study, we found that FOXP1 is upregulated in prolactinoma samples and presented a negative expression association with CLRN1-AS1. Further mechanism study revealed that FOXP1 transcriptionally suppressed CLRN1-AS1 in pituitary prolactinoma. Molecular mechanism by which FOXP1 was upregulated in prolactinoma samples will be investigated in our future study. In conclusion, our study revealed that FOXP1-induced CLRN1-AS1 modulates cell growth and autophagy in pituitary prolactinoma by regulating miR-217/DKK1 axis to inactivate the Wnt/β-catenin signaling pathway. Our research findings may provide a new prospective on the molecular research in pituitary prolactinoma.

## Supplementary information

Supplementary Figure 1

Supplementary Figure 2

Supplementary Figure 3

supplementary figure legends
