# Supplementary figures and images for: FOXP1-induced lncRNA CLRN1-AS1 acts as a tumor suppressor in pituitary prolactinoma by repressing the autophagy via inactivating Wnt/β-catenin signaling pathway
Source: Cell Death Dis. 2019 Jun 24;10(7):499. doi: 10.1038/s41419-019-1694-y (PMC6591247; doi:10.1038/s41419-019-1694-y)

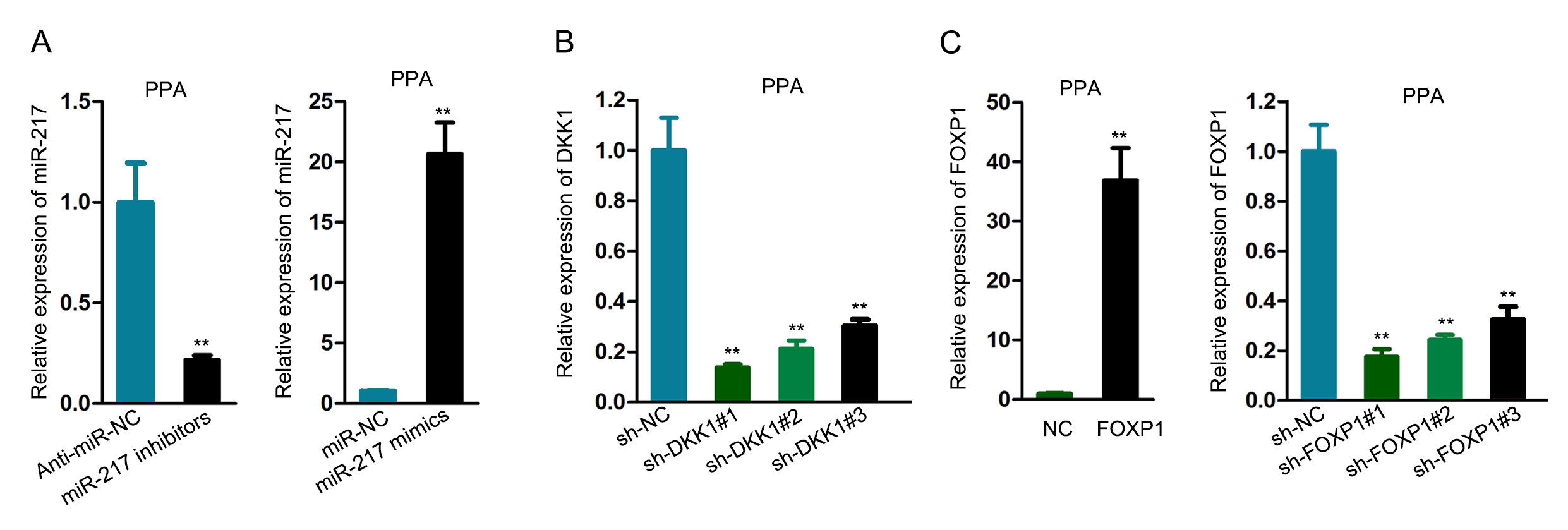

Supplement: Supplementary file 1 — Supplementary Figure 1 [file 41419_2019_1694_MOESM1_ESM.tif]

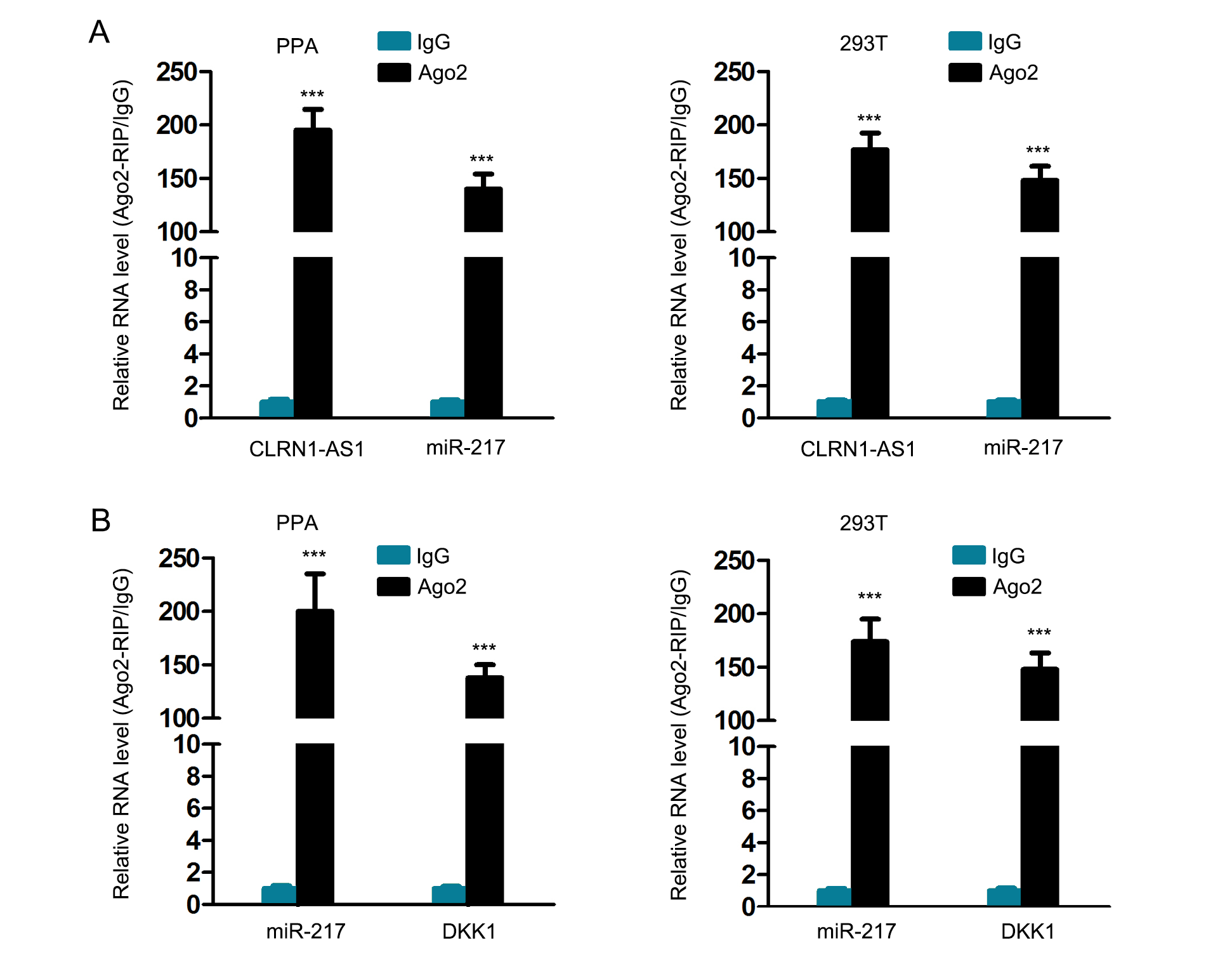

Supplement: Supplementary file 2 — Supplementary Figure 2 [file 41419_2019_1694_MOESM2_ESM.tif]

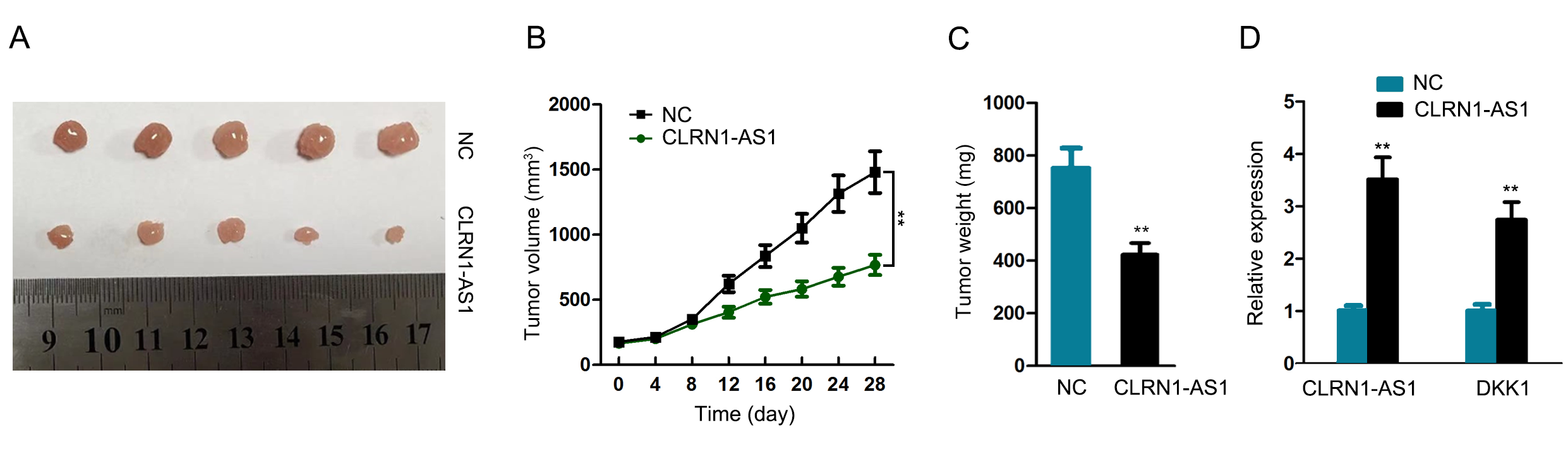

Supplement: Supplementary file 3 — Supplementary Figure 3 [file 41419_2019_1694_MOESM3_ESM.tif]
