# Supplementary material for: FOXP1-induced lncRNA CLRN1-AS1 acts as a tumor suppressor in pituitary prolactinoma by repressing the autophagy via inactivating Wnt/β-catenin signaling pathway
Source: Cell Death Dis. 2019 Jun 24;10(7):499. doi: 10.1038/s41419-019-1694-y (PMC6591247; doi:10.1038/s41419-019-1694-y)
Supplement: Supplementary file 4 — supplementary figure legends [file 41419_2019_1694_MOESM4_ESM.docx]

**Supplementary Figure 1. A.** miR-217 expression was increased or decreased by transfecting with miR-217 mimics or inhibitors. **B.** Knockdown efficiency for DKK1. **C.** FOXP1 was overexpressed in PPA cell and was silenced in PPA cell. ^**^P < 0.01 vs control group, indicated data are statistically significant.

**Supplementary Figure 2. A-B.** Interaction between CLRN1-AS1 and miR-217 as well as between miR-217 and DKK1 was demonstrated by Ago2-RIP assay. ^***^P < 0.001 vs control group, indicated data are statistically significant.

**Supplementary Figure 3. CLRN1-AS1 suppressed cell growth in vivo. A.** Tumors derived from PPA cells transfected with pcDNA-CLRN1-AS1 or empty vector. **B-C.** Tumor volume and tumor weight in different groups were calculated. D. The expression of CLRN1-AS1 and DKK1 in response to CLRN1-AS1 overexpression. ^**^P < 0.01 vs control group, indicated data are statistically significant.
